# Information distribution on regions of speckle patterns for imaging of multimode fiber

**DOI:** 10.1016/j.heliyon.2023.e13357

**Published:** 2023-02-01

**Authors:** Shenyi Liu, Yunxu Sun, Wei Liu, FuChen Xiao, Haoyang Song

**Affiliations:** School of Electronics and Information Engineering, Harbin Institute of Technology, Shenzhen 518055, China

**Keywords:** Multi-mode fiber, Imaging, Neural network, Region segmentation

## Abstract

Multimode fibers (MMF) have been extensively investigated for transmitting images. The transmitting images are distorted into speckle patterns by MMFs, which can be reconstructed by neural networks. We studied the information distribution of MMF speckle patterns for image reconstruction. The speckle patterns, segmented by three methods of segmentation, as Centering (1), Quartering (2) and Surrounding (3), are reconstructed into input images by Complex Artificial Neural Network (CANN). Experimental results show that only about one third of full speckle patterns is enough to reconstruct the original images. The quality of reconstructed image is related to the cropping method with different frequency components in speckle patterns, under the same cropped size, Centering segmentation has 4% performance improvement compared to Surrounding segmentation. Optimized segmentation will improve the quality of reconstructed images.

## 1 Introduction

Endoscopic imaging is typically based on single-mode fiber bundles or hybrid systems of fiber optics and mechanical actuator [1]. The cross section of these devices typically ranges from half a millimeter up to a few millimeters, which makes them unsuitable for some biological applications such as in vivo imaging of neural activity. Multi-mode optical Fibers (MMF) have multiple guiding modes that can transmit information in parallel at the same time, and can realize wide field imaging independently. MMFs have the advantages of a small diameter with tens of microns and the ability of bending at a small angle. MMFs, as an image transmission device, can be the minimized imaging probe of ultra-thin endoscopes with high resolution [2], which are promising for non-invasive diagnosis, minimally invasive surgery and non-invasive surgery. However, the dispersions of MMFs distort the transmitted image into speckles. Therefore, the main purpose of the research of MMFs is to eliminate or to compensate the distortion caused by mode dispersion and mode coupling of MMFs, which requires the knowledge of the transmission matrix of MMF.

The transmission matrix is complex, which needs both the amplitude and the phase of the output patterns corresponding to those of the input patterns [[3], [4], [5], [6]]. A lot of measurements and calculations are required to construct the transmission matrix, commonly. A variety of new ideas using wavefront shaping techniques [3,4,[7], [8], [9]] have demonstrated the possibility of controlling the propagation of these modes, showing their imaging potential. Several groups have developed ultrathin endoscopes based on MMFs and wavefront shaping for a wide range of biomedical imaging modalities including wide-field microscopy [3], confocal [6] and Photoacoustic imaging [10,11]. However, these methods require external reference beam to extract the complex optical field (amplitude and phase) by interference, which results in complicated experiments.

The ideas of using neural networks on MMFs has been proposed for almost three decades [[12], [13], [14]]. As for the ubiquitous availability of computing power through graphical processing units and new types of neural network architectures, applications of neural networks are reviving [15]. The neural network learns the relationship between input and output of samples and adaptively adjusts the transmission matrix through training samples, which does not need a lot of measurement and calculation, but only needs amplitude information to fit the transmission matrix. Accordingly the structures of imaging system with MMFs are simple [16]. However, the computational complexity of neural network is closely related to the size of input data. The larger the size of feature graph, the more parameters are needed to construct the neural network.

The input lights are decomposed into separate guiding modes to transmit through the MMFs. Therefore, the speckle patterns at distal end of MMFs contain the spatial characteristics information. Understanding the distribution characteristics of speckle patterns is meaningful, which may effectively reduce the calculation of redundant information in the training of neural networks. If only part of speckle information can be used to reconstruct the original image, the speed of image reconstruction will be enhanced. Accordingly, the time costs will be reduced.

In this paper, the cropped speckle patterns in different areas from the original are used for image reconstruction through neural network. The distribution of original image information in speckle patterns is studied, part of speckle information can be utilized for reconstruction, which is intuitive for the accurate and real-time imaging of MMFs based on neural network.

## 2 Methods

### 2.1 Theoretical analysis

MMF imaging experiments are demonstrated with step-index MMFs. Through MMFs, the relation between the input light field $U_{o}$ at the proximal end and the output light field $U_{i}$ at the image plane is described as

(1) $$U_{o}=\sum_{u=0}\sum_{v=0}A_{uv}\left(x_{1},y_{1}\right)\varepsilon_{uv}\left(x_{1},y_{1}\right)$$

and

(2) $$\begin{array}{l} U_{i}\left(\eta ,\xi \right)=\sum_{u=0}\sum_{v=0}\iint sA_{uv}\left(x_{1},y_{1}\right)\varepsilon_{uv}\left(x_{1},y_{1}\right)\exp\left(-j\beta_{uv}Z_{L}\right)\\ \exp\left(\frac{jk}{2Z_{i}}\left[{\left(\eta -x_{L}\right)}^{2}+{\left(\xi -y_{L}\right)}^{2}\right]\right)dx_{L}dy_{L} \end{array}$$

where $\varepsilon_{uv}$ is the transverse mode with radial index *u* and angular index *v* respectively, $A_{uv}$ is the amplitude of the mode, $\beta_{uv}$ is the propagation constant, $Z_{L}$ is the length of MMF. The sum terms represent the coupling between the input light field and the guiding modes, and the integral terms represent the free space Fresnel diffraction process from the distal end to the image plane.

The digital images can be regarded as discrete pixel matrix, which are stored as one-dimensional vectors for simplicity. The coordinates of object plane and image plane are $n_{o}$ and $n_{i}$. Eq. (1) and Eq. (2) can be rewritten as

(3) $$U_{i}\left(n_{i}\right)=h_{oi}\left(n_{i},n_{o}\right)⊗U_{o}\left(n_{o}\right)$$

where $h_{oi}$ is the point spread function (PSF) from proximal end $Z_{o}$ to the image plane $Z_{i}$.

In eq. (3), we can see that the process of light transmission in MMFs can be described by point spread function

(4) $$U_{i}\left(n_{i}\right)=\int_{-\infty }^{\infty }h\left(n_{o};n_{i}\right)U_{o}\left(n_{o}\right){dn}_{o}$$

After transmitting in MMFs, the light waves on each object point are mixed with each other, and the image plane is completely distorted. At the same time, each object point contributes to all image points on the image plane.

### 2.2 Experimental setup

**Fig. 1:**
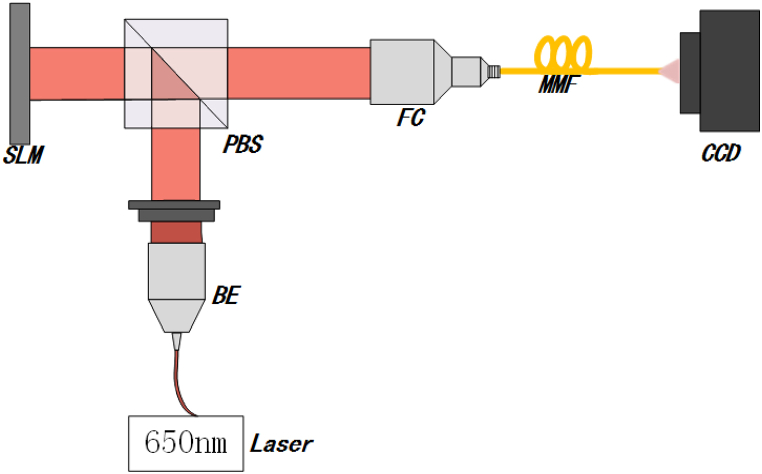
Apparatus of the experimental setup. Laser: 650 nm, RealLight, AWS-650-ISF-002; BE: beam expander; PBS: polarization beam splitter; SLM: spatial light modulator, Cas Microstar; FC: doublet collimator; MMF: 0.75 m length, 62.5 μm core diameter, 0.275 NA.

The single multimode fiber image transmission system is built on an air floating vibration isolation platform. To avoid external stray light interference and air disturbance, the experimental platform is shielded by a shading cloth. The entire experimental system is illustrated in Fig. 1.

A laser beam with a wavelength of 650 nm, generated by a semiconductor laser (RealLight, AWS-650-ISF-002), is collimated to a beam with a diameter of 7 mm by a telescope and is split by a Polarization Beam splitter (PBS). The reflected beam illuminates on Spatial light Modulator (SLM) that reflects the light with image information as the object light for imaging. The object light is coupled into single MMF by a fiber coupler (FC). After transmitting through the MMF, the emitted light forms the speckles, recorded by a charge couple device (CCD).

### 2.3 Algorithms

The transmission of light wave in MMFs is commonly described by complex functions in mathematics. To improve the fitting accuracy with the same number of hidden layers compared to traditional ANN networks, and to be able to implement equivalent complex operations in real neural networks with less original information lost. We need to change the node values and weight parameters of traditional networks in the form of real values, equivalent complex neurons are represented from that of real numbers in neural network. Followingly, Complex Artificial Neural Network (CANN) is designed on the basis of single-layer fully connected layer Neural Network [17]. In form, the equivalent complex neurons are represented by real numbers, the real and imaginary part of a complex number can be represented by two different real components, assuming that there are N feature graphs in a certain layer, another N feature graphs are initialized to ensure the total number of graphs is a multiple of two. In order to represent each element of the feature graphs as a complex number, the first N feature graphs are as the real part and the last N feature graphs are as the imaginary part of the complex number, and then the computation rules of Neural Network are redefined to realize equivalent complex operation by convolution between complex feature graphs and complex weight tensors.

### 2.4 Region segmentation

In order to fit the transmission matrix, which is closer to the real transmission characteristics of optical fiber, it is necessary to train the neural network with a large amount of data with different features. In this paper, the open-source ImageNet natural scene sample set are loaded on SLM for training. The structural features of ImageNet set are complicated, which can ensure the feasibility of training. The speckle pattern resolution of the ImageNet set is set as 120 × 120 pixels.

There are three methods of segmentation, as Centering (1), Quartering (2) and Surrounding (3), to study the distribution of effective information in different regions of speckle patterns.

**Fig. 2:**
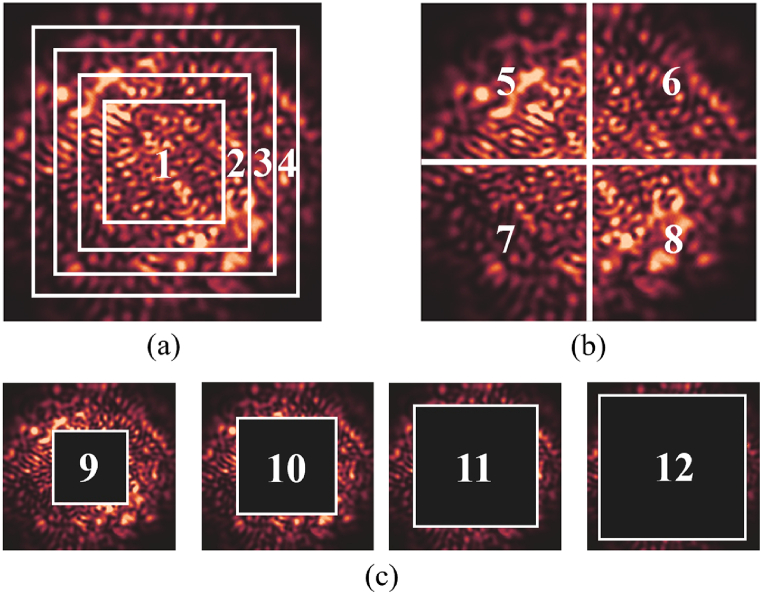
Region Segmentation methods (a) Centering (1); (b) Quartering (2); (c) Surrounding (3).

Centering (1), shown in Fig. 2(a), crops the speckle patterns with different side lengths from the center to the outside, which are named as region 1 to 4;

Quartering (2), shown in Fig. 2(b), transfers the cropped location from center to the corner, dividing the speckle pattern into four regions with the same area, named as region 5 to 8;

Surrounding (3), shown in Fig. 2(c), removes the central region of the speckle patterns, leaving only the surrounding information. With different removal areas, the cropped regions are named as region 9 to 12.

For the speckle patterns with resolution of 120 × 120 pixels imaged by ImageNet natural scene sample set, the cropped sizes of region 1 to 4 are set as 40 × 40, 60 × 60, 80 × 80 and 100 × 100 pixels, respectively. The cropped size of region 5 to 8 are all set as 60 × 60 pixels. And the resolution of speckle shielding areas of region 9 to 12 are set as 60 × 60, 70 × 70, 80 × 80 and 90 × 90 pixels, respectively.

## 3 Experiment results

**Fig. 3:**
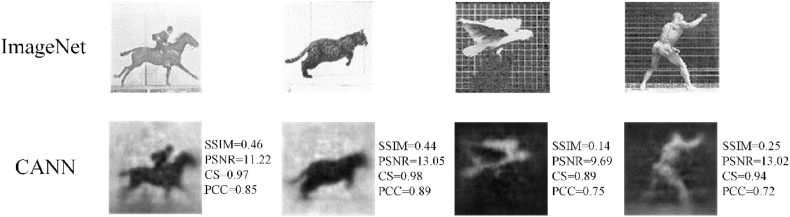
Reconstruction results with full speckle patterns. Row 1: Original images from ImageNet set. Row 2: Reconstruction results.

Based on the MMF imaging setup in Fig. 1, CANN neural networks are trained with the data set made of full speckle patterns, the training set used to train the neural network consists of 5000 speckle images and 5000 images of ImageNet natural scene set corresponding to speckles. 10% of the image pairs are randomly selected from the training set and used as verification set. In addition, 500 speckle image pairs were selected as the test set. The images in the test set were not used in the training process and were only used to test the recovery effect of the network on unknown speckle patterns. We test the feasibility of the trained CANN network, by reconstructing four images of ImageNet natural scene set from the speckles, shown in Fig. 3.

Based on the MMF imaging setup in Fig. 1, CANN neural networks are trained with the data set made of full speckle patterns. We test the feasibility of the trained CANN network, by reconstructing four images of ImageNet natural scene set from the speckles, shown in Fig. 3.

**Fig. 4:**
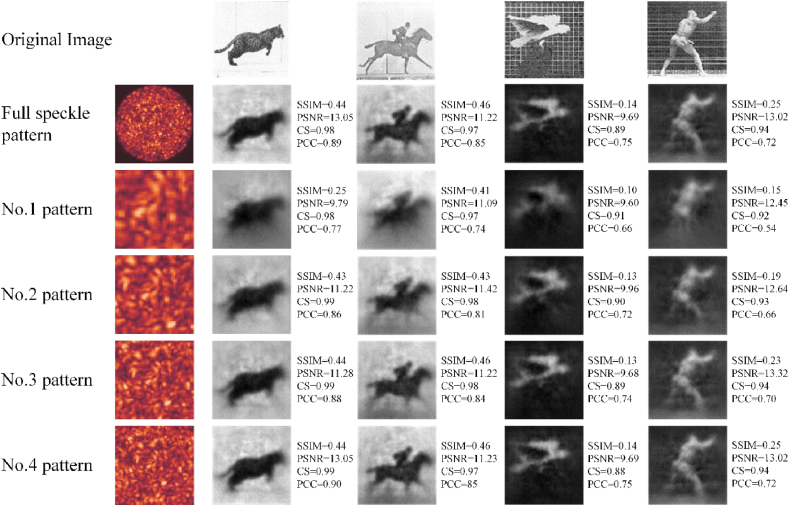
Reconstruction results with speckle patterns of the Centering (1). Column 1: speckle patterns with different cropped size. Column 2–5: Reconstruction results of four datasets. SSIM, PSNR, CS and PCC are average of test set, not the value of a single image.

**Fig. 5:**
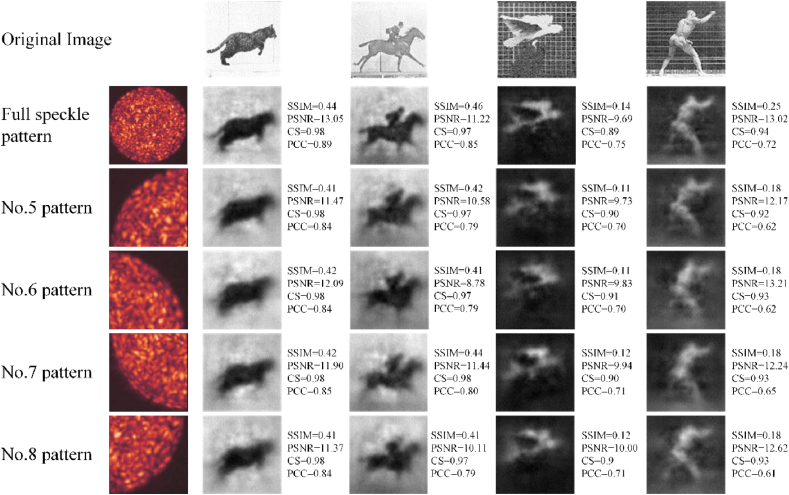
Reconstruction results with speckle patterns cropped from the corner. Column 1: speckle patterns with different cropped location. Column 2–5: Reconstruction results of four datasets.

**Fig. 6:**
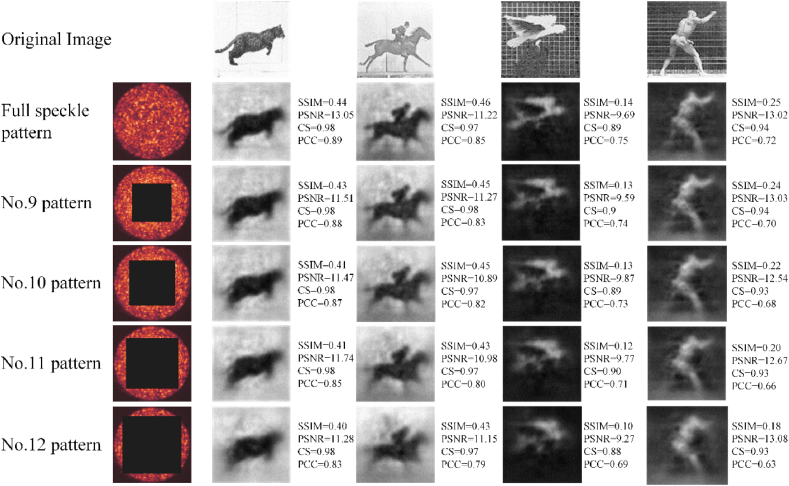
Reconstruction results of speckle patterns with center shielded. Column 1: speckle patterns with different shielding size. Column 2–5: Reconstruction results of four datasets.

The speckle patterns are cropped by the above three methods in Section 2.4, respectively. Trained with the cropped speckle patterns, CANN successfully reconstructs the images, shown in Fig. 4, Fig. 5, Fig. 6. The quality of reconstruction is characterized by structural similarity coefficient (SSIM), peak signal-to-noise ratio (PSNR), cosine similarity (CS) and Pearson correlation coefficient (PCC).

The reconstruction results of the Centering (1) are shown in Fig. 4. The quality of reconstruction decreases with decreasing cropped size. As the cropped size from inside to outside increases gradually, the corresponding image quality gets closer to the reconstruction quality of the uncropped. For a cropped size of 40 × 40 pixels of region 1, equivalent to around 11.1% area of the full speckle pattern, the SSIM is decreased by 0.096. The images are reconstructed with a low signal-to noise ratio, which only displays blurred information and is difficult for humans to discriminate the objects in images. Meanwhile, the IQA of reconstructed images corresponding to cropped speckle pattern 3 and 4 is the same or even better than that of the original speckle pattern reconstruction.

From eq. (3) and eq. (4), we can see that the process of image transmission in the MMF can be represented by the PSF of the MMF. Any point on the input plane contributes different weights to the points on the output plane, that is, each speckle pattern contains the information of the whole image.

The reconstruction results of Quartering (2) are shown in Fig. 5, where the CANN is trained successfully to reconstruct the whole images. The reconstruction results are only different in details. e.g., the image of “the jumping cat”, the range of SSIM is only 0.01 and all four reconstructed images have the same CS.

Fig. 6 shows that in the case of Surrounding (2), the tested images can also be reconstructed, with some detail features lost. Comparing the reconstruction effect of region 9, there is almost no difference in the main part of the image “Jumping cat” in subjective vision. However, the background noise increases, and the PSNR deteriorates. When the removing size grows larger, the image quality gets worse. The overall resolution of the pattern has not changed compared to the original speckle pattern, but the speckles available to extract image information decreases significantly. For the removing size of 90 × 90 pixels, equivalent to around 28% of the full speckle pattern, the background noise is significant, with the main part of images being retained.

## 4 Discussion

**Fig. 7:**
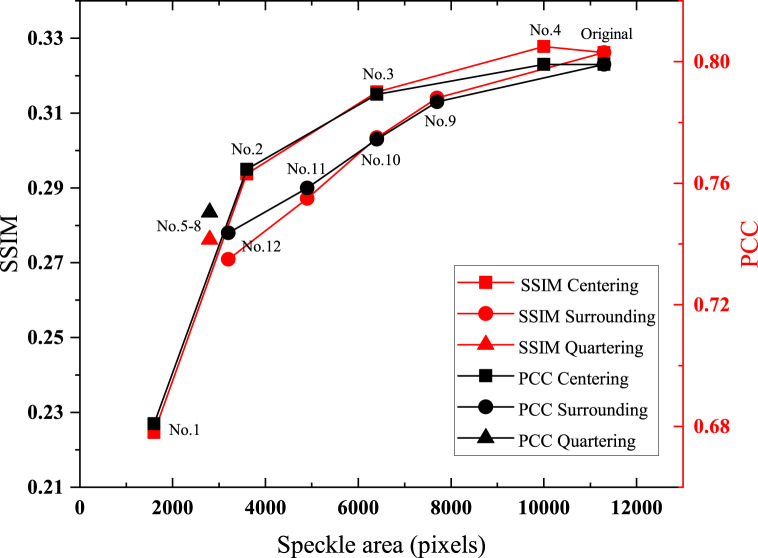
Relationship between the available speckle area (in pixels) and the image quality.

To further investigate the information distribution in different cropped regions. Fig. 7 shows the relationship between image quality and available speckle areas in different segmentation methods. And the image quality assessment (IQA) of reconstruction in different regions is shown in Table 1.

**Table 1:** IQA for speckle pattern reconstruction of ImageNet with CANN.

Cropped pattern	Available pixels	SSIM	PCC
1	1600	0.227	0.678
2	3600	0.295	0.763
3	6400	0.315	0.790
4	10000	0.323	0.805
5	2800	0.280	0.737
6	2800	0.286	0.738
7	2800	0.287	0.753
8	2800	0.281	0.738
9	7700	0.313	0.788
10	6400	0.303	0.775
11	4900	0.290	0.755
12	3200	0.278	0.735
Original pattern	11300	0.323	0.803

The experimental results demonstrate that the information, carried by cropped speckle patterns, is almost proportional to the available speckle area, which can be seen from the improvements of IQA in Table 1. But comparing the image quality of region 3 and 4 with that of full speckle patterns, the IQA with fewer available pixels stays the same and even gets better. There are less noises with high frequency components in the center region compared to the surrounding region on the original speckle patterns. The original image information on speckle patterns has redundancy. It is not necessary to reconstruct using full speckle pattern. A part of the information is sufficient.

The results of image reconstruction using speckle patterns cropped by Quartering segmentation is almost the same, which can also be reflected from IQA. The four regions from different quadrants play almost the same role in image reconstruction tasks. To further illustrate the influence of cropped location on image reconstruction effect, we compared the image quality using region 5 to 8 with that using region 2, and all the five cropped speckle patterns have the same resolution of 60 × 60 pixels. The image quality of the cropped speckle patterns from the quadrants is slightly lower than that from the center, which can be explained by the different speckle information that is available for training neural networks. We can see that the energy of the target image used for reconstruction is mainly concentrated in the low-frequency region, which corresponds to the low-frequency components distributed in the central region of speckle patterns. Besides, not all the cropped regions of region 5 to 8 contain image information, only a quarter of the circle is available to extract information. There is a difference in effective area of $w^{2}-0.25\pi w^{2}$ between region 2 and region 5 to 8, where w is the side length of the region delimited, resulting in the difference of reconstruction effect.

In Fig. 7, we show the trending of SSIM and PCC of the reconstructed images of Centering (1) and Surrounding (3). The difference of these two methods shows that the location of the cropped speckle patterns influences the image quality. Centering (1) has about 4% improvement on reconstruction quality compared to Surrounding (3) under the same available speckle area. The above phenomenon shows that the final reconstruction results can be influenced by both cropped size and frequency component distribution (cropped location), with the cropped size plays a more important role.

The reconstruction quality of full original speckle patterns is slightly lower than that of Centering (1) with cropped pattern 4. This phenomenon can be explained by the distribution of information from different frequency components in full speckle patterns. The noises with high frequency components are concentrated in the edge of the original speckle patterns, which affects the image reconstruction in this part of the area. As the available area of speckle patterns reducing, the edge contour of the main body of the reconstructed image is still well preserved. Meanwhile, because of removing the region with more noises, the reconstruction quality of Centering (1) is better than that of full speckle patterns. The phenomena further indicate that the low-frequency components of the image are mainly concentrated at the center of the speckle patterns (image information), while the high-frequency components are mainly concentrated at the edge of the speckle patterns.

The larger the proportion of the input speckle pattern to the original pattern, the more available feature information can be used to enhance the quality of image reconstruction. But in Fig. 7 we can see that about one third of the speckle area is enough to achieve acceptable image reconstruction quality. When the speckle area keeps getting smaller, the reconstruction quality decreases sharply.

## 5 Conclusion

In this paper, we study the original image information carried by the local speckles in different locations. The training set and test set are made by cropped speckle patterns from different locations of the original speckle patterns. The original image is reconstructed successfully by neural network. The results show that the reconstruction effect is mainly related to the available speckle size. The smaller the speckle size as the input, the smaller the number of parameters and computation required by neural network. However, it is worth mentioning that there are frequency components that differ in different speckle locations. By cropping the original speckle pattern in a proper way with the knowledge of frequency distribution in target image, we can effectively reduce the time complexity of network algorithm, and the time required for single speckle reconstruction, which is of great significance for the development of endoscopy real-time video imaging.

## Author contribution statement

Shenyi Liu: Analyzed and interpreted the data; Contributed reagents, materials, analysis tools or data; Wrote the paper.

Yunxu Sun: Conceived and designed the experiments; Analyzed and interpreted the data; Wrote the paper.

Wei Liu: Analyzed and interpreted the data; Contributed reagents, materials, analysis tools or data.

FuChen Xiao: Conceived and designed the experiments; Performed the experiments.

Haoyang Song: Performed the experiments.

## Funding statement

Yunxu Sun was supported by Science and Technology Planning Project of Shenzhen Municipality [JCYJ20190806142610885].

## Data availability statement

Data will be made available on request.

## Declaration of interest’s statement

The authors declare no competing interests
